# Supplementary material for: Class A and B GPCRs trigger rapid Gαs translocation to late and slow recycling endosomes
Source: Commun Biol. 2025 Nov 28;8:1715. doi: 10.1038/s42003-025-09117-1 (PMC12663142; doi:10.1038/s42003-025-09117-1)
Supplement: Supplementary file 3 — Description of Additional Supplementary files [file 42003_2025_9117_MOESM3_ESM.pdf]

Description of Additional Supplementary files

File name: Supplementary Data 1

Description: Source data used for generating the plots in the main figures are available in the Supplementary Data.
