# Supplementary material for: Class A and B GPCRs trigger rapid Gαs translocation to late and slow recycling endosomes
Source: Commun Biol. 2025 Nov 28;8:1715. doi: 10.1038/s42003-025-09117-1 (PMC12663142; doi:10.1038/s42003-025-09117-1)
Supplement: Supplementary file 5 — Reporting summary [file 42003_2025_9117_MOESM5_ESM.pdf]

nature portfolio

Corresponding author(s): Christine Lavoie

Last updated by author(s): Oct 6, 2025

Reporting Summary

Nature Portfolio wishes to improve the reproducibility of the work that we publish. This form provides structure for consistency and transparency in reporting. For further information on Nature Portfolio policies, see our Editorial Policies and the Editorial Policy Checklist.

Statistics

For all statistical analyses, confirm that the following items are present in the figure legend, table legend, main text, or Methods section.

n/a	Confirmed	
☐	☒	The exact sample size (*n*) for each experimental group/condition, given as a discrete number and unit of measurement
☐	☒	A statement on whether measurements were taken from distinct samples or whether the same sample was measured repeatedly
☐	☒	The statistical test(s) used AND whether they are one- or two-sided<br>*Only common tests should be described solely by name; describe more complex techniques in the Methods section.*
☒	☐	A description of all covariates tested
☒	☐	A description of any assumptions or corrections, such as tests of normality and adjustment for multiple comparisons
☐	☒	A full description of the statistical parameters including central tendency (e.g. means) or other basic estimates (e.g. regression coefficient) AND variation (e.g. standard deviation) or associated estimates of uncertainty (e.g. confidence intervals)
☒	☐	For null hypothesis testing, the test statistic (e.g. *F*, *t*, *r*) with confidence intervals, effect sizes, degrees of freedom and *P* value noted<br>*Give P values as exact values whenever suitable.*
☒	☐	For Bayesian analysis, information on the choice of priors and Markov chain Monte Carlo settings
☒	☐	For hierarchical and complex designs, identification of the appropriate level for tests and full reporting of outcomes
☒	☐	Estimates of effect sizes (e.g. Cohen's *d*, Pearson's *r*), indicating how they were calculated

Our web collection on statistics for biologists contains articles on many of the points above.

Software and code

Policy information about availability of computer code

Data collection	BRET signal were measured on a TriStar2 LB 942 Multimode Reader (Berthold Technologies). LAS AF Lite software (Leica) was used for image acquisition and analysis.
Data analysis	For BRET experiments, data were analyzed in GraphPad Prism 10.5 software. The confocal images were further processed using Adobe Photoshop v 26.8.1 (Adobe Systems). Huygens professional 23.10 was used for colocalization quantification using Mander's overlap. coefficient.

For manuscripts utilizing custom algorithms or software that are central to the research but not yet described in published literature, software must be made available to editors and reviewers. We strongly encourage code deposition in a community repository (e.g. GitHub). See the Nature Portfolio guidelines for submitting code & software for further information.

Data

Policy information about availability of data

All manuscripts must include a data availability statement. This statement should provide the following information, where applicable:

- Accession codes, unique identifiers, or web links for publicly available datasets
- A description of any restrictions on data availability
- For clinical datasets or third party data, please ensure that the statement adheres to our policy

All relevant data supporting the findings are available within the paper and the Supplementary Materials. Additional information and reagents are available from the corresponding author upon request.

Research involving human participants, their data, or biological material

Policy information about studies with human participants or human data. See also policy information about sex, gender (identity/presentation), and sexual orientation and race, ethnicity and racism.

Reporting on sex and gender	N/A
Reporting on race, ethnicity, or other socially relevant groupings	N/A
Population characteristics	N/A
Recruitment	N/A
Ethics oversight	N/A

Note that full information on the approval of the study protocol must also be provided in the manuscript.

Field-specific reporting

Please select the one below that is the best fit for your research. If you are not sure, read the appropriate sections before making your selection.

☒ Life sciences ☐ Behavioural & social sciences ☐ Ecological, evolutionary & environmental sciences

For a reference copy of the document with all sections, see nature.com/documents/nr-reporting-summary-flat.pdf

Life sciences study design

All studies must disclose on these points even when the disclosure is negative.

Sample size	At least three biological replicates and clearly stated within the figure legends.
Data exclusions	No data were excluded from the analysis.
Replication	All attempts at replication were successful. Individual experiments (n) were performed on different days from different transfections. At least three bilogical replicates were carried ou for each experiment.
Randomization	Experiments were not randomized.
Blinding	The investigators were not blinded.

Reporting for specific materials, systems and methods

We require information from authors about some types of materials, experimental systems and methods used in many studies. Here, indicate whether each material, system or method listed is relevant to your study. If you are not sure if a list item applies to your research, read the appropriate section before selecting a response.

Materials & experimental systems

n/a	Involved in the study
☐	☒ Antibodies
☐	☒ Eukaryotic cell lines
☒	☐ Palaeontology and archaeology
☒	☐ Animals and other organisms
☒	☐ Clinical data
☒	☐ Dual use research of concern
☒	☐ Plants

Methods

n/a	Involved in the study
☒	☐ ChIP-seq
☒	☐ Flow cytometry
☒	☐ MRI-based neuroimaging

Antibodies

Antibodies used: Anti-Myc mouse (#2276) , Anti-EEA1 rabbit (#3288) from Cell Signaling; Anti-HA rabbit (#PRB-101P) from Covance; Alexa Fluor 594 against mouse (#A21203), Alexa Fluor 594 against rabbit (#A21207), Alexa Fluor 488 against mouse (#A21202) Alexa Fluor 488 against rabbit (#A21206) from Invitrogen, Anti-G alpha S-HRP (#sc-135914-HRP) from Santa Cruz, HRP anti-rabbit from Bio-Rad.

Validation: Validated by the provider and used in many published studies

Eukaryotic cell lines

Policy information about cell lines and Sex and Gender in Research

Cell line source(s): HEK293SL cells were kindly provided by Dr. Stéphane Laporte (McGill University, Montreal, Quebec, Canada). HEK293T-Gαs KO (HEK293T Gαs crispr knock-out) cell lines were kindly provided by Dr Asuka Inoue (Tohoku University, Sendai, Miyagi, Japan).

Authentication: Cell lines were authenticated by western blot to determine Gαs knockout.

Mycoplasma contamination: Cells were regularly tested for mycoplasma contamination (PCR Mycoplasma Detection kit) and only mycoplasm-free cells were used.

Commonly misidentified lines (See ICLAC register): N/A

Plants

Seed stocks: N/A

Novel plant genotypes: N/A

Authentication: N/A
