# Supplementary material for: Class A and B GPCRs trigger rapid Gαs translocation to late and slow recycling endosomes
Source: Commun Biol. 2025 Nov 28;8:1715. doi: 10.1038/s42003-025-09117-1 (PMC12663142; doi:10.1038/s42003-025-09117-1)
Supplement: Supplementary file 2 — Supplementary Information [file 42003_2025_9117_MOESM2_ESM.pdf]

Supplementary information

1. Supporting methods

Confocal microscopy
Confocal microscopy analyses were done as described in the Experimental section of the article.

BRET measurements
BRET experiments were done as described in the Experimental section of the article.

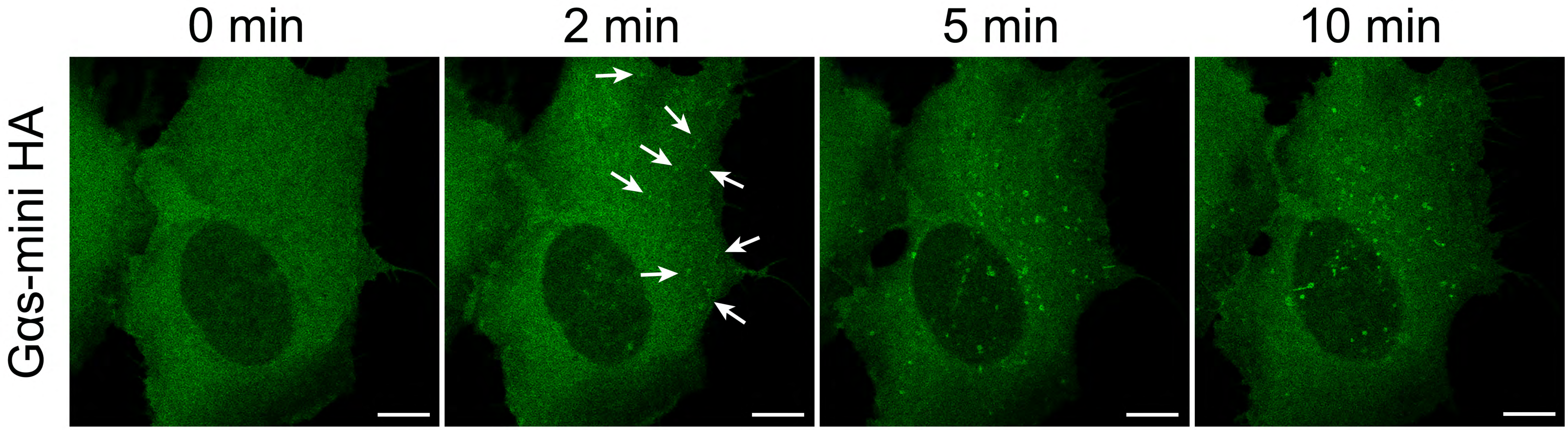

Supplementary Figure 1. EGFP Tag Does Not Alter Gαs Trafficking. Serum-starved HEK293 cells co-expressing Flag-β2AR, Gαs-mini HA, and frankenbody-EGFP were stimulated with 10 μM isoproterenol (Iso) and visualized by live-cell confocal microscopy. Arrows highlight representative Gαs-positive vesicles apparent at early time points. Scale bars, 10 μm.

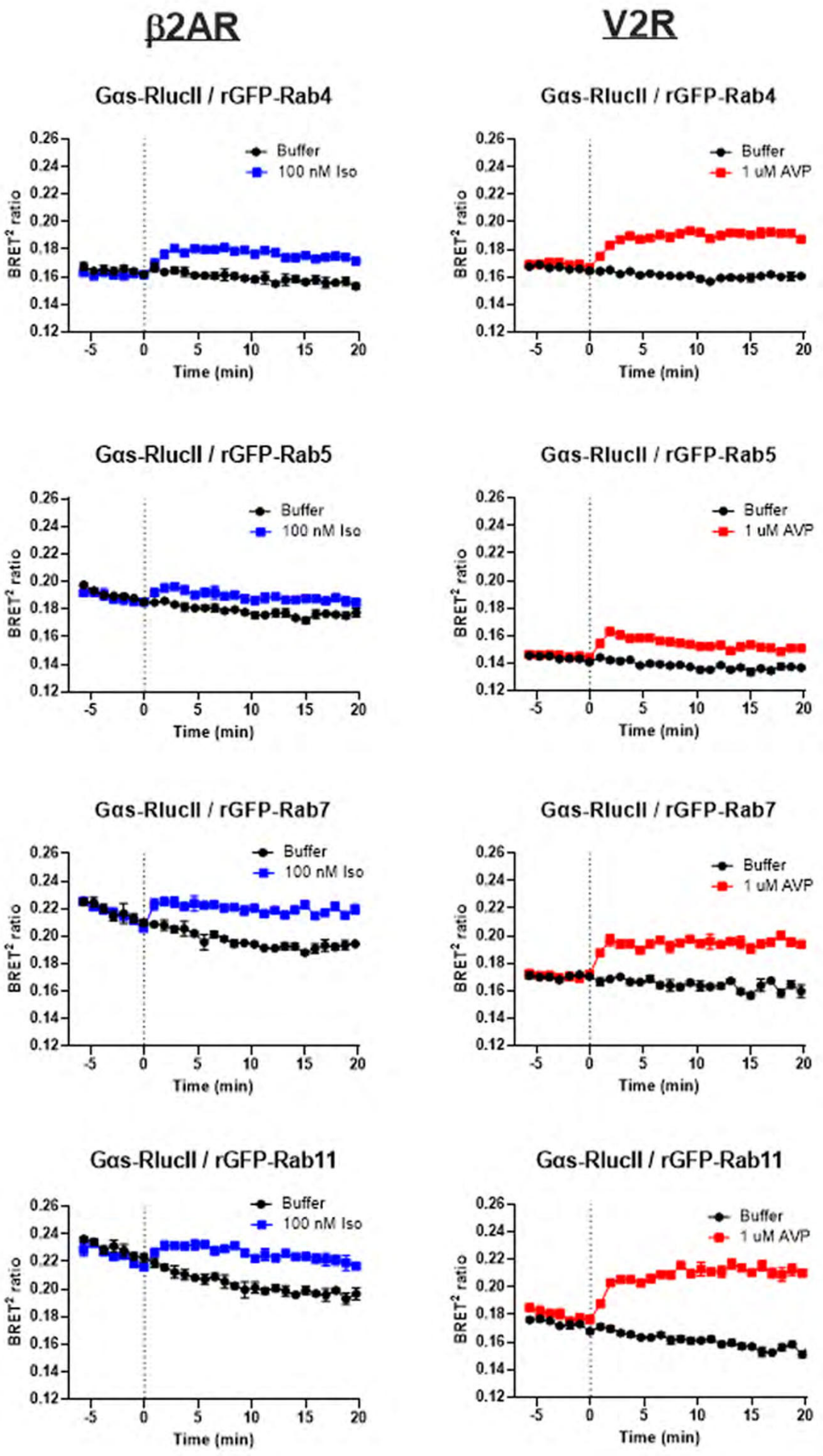

Supplementary Figure 2. Gαs Recruitment to Different Endosomal Compartments Following β2AR or V2R Activation (complementary to Figure 5B). Time courses of Gαs translocation to Rab4-, Rab5-, Rab7-, and Rab11-labeled endosomes were measured following stimulation with 1 µM Iso (β2AR agonist) or 1 µM AVP (V2R agonist), using the ebBRET biosensor assay described in Figure 5A. The vertical dotted line indicates the timing of agonist addition. Data are presented as mean ± SEM of triplicates from a representative experiment; experiments were performed three times with similar results.

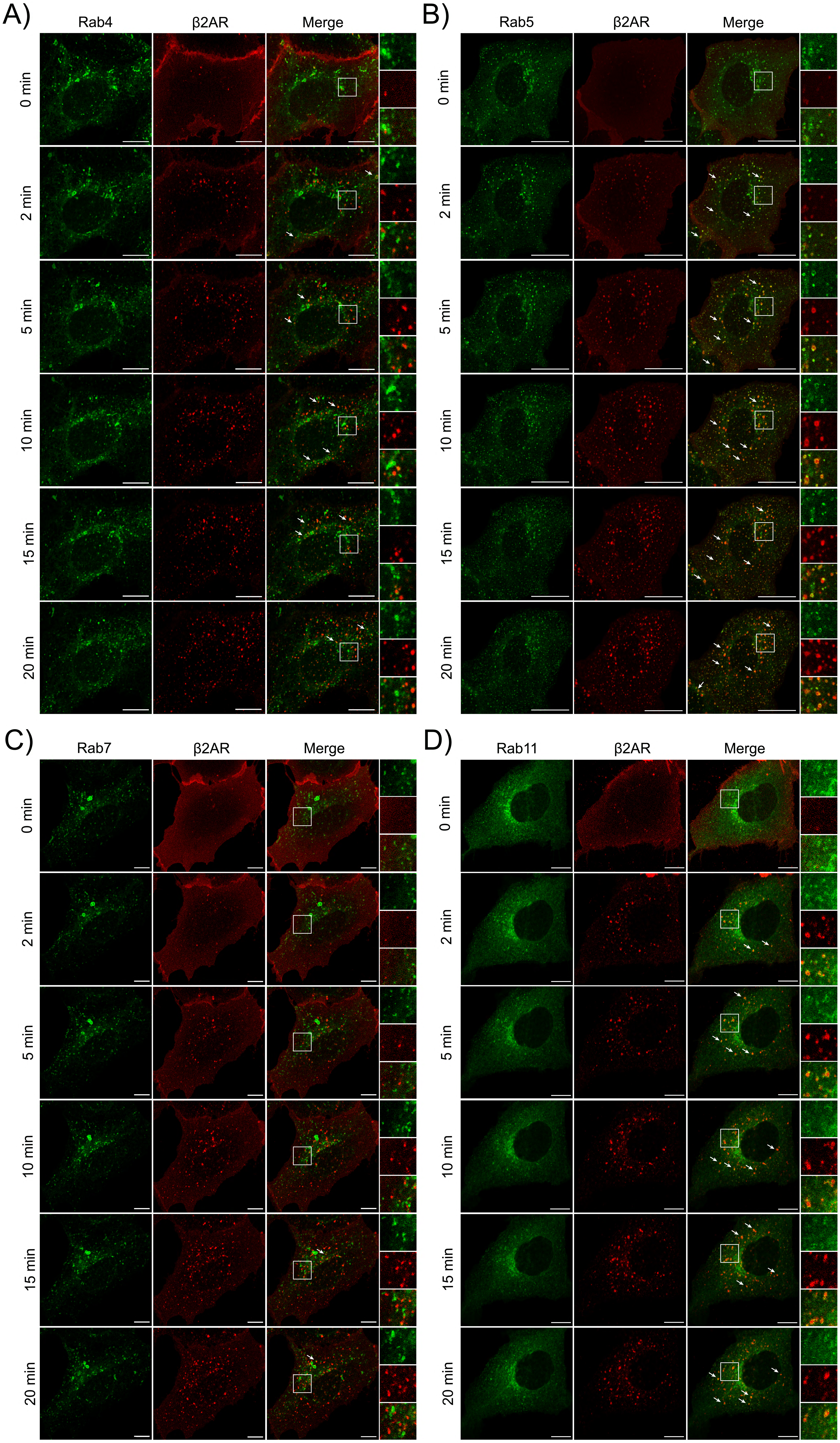

Supplementary Figure 3. Endosomal Distribution of β2AR. Live-cell confocal imaging of serum-starved HEK293 cells co-expressing SNAP-β2AR and Tdr-rGFP-tagged endosomal markers: (A) Rab4, (B) Rab5, (C) Rab7, or (D) Rab11. Cells were stimulated with 10 μM isoproterenol (Iso) for 20 min at 37°C. Arrows indicate representative sites of colocalization between β2AR and Rab-marked endosomes. Scale bars, 10 μm.

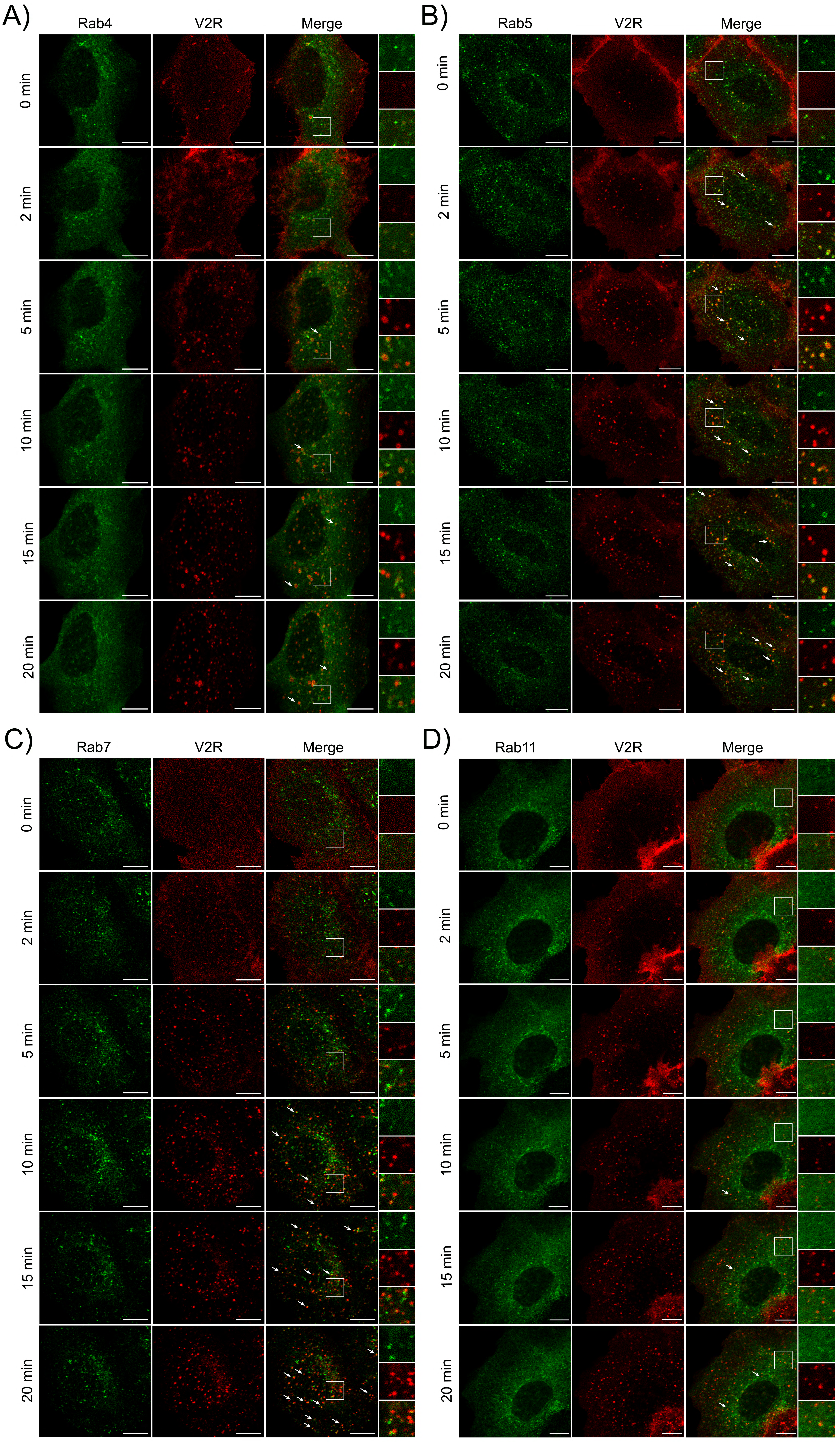

Supplementary Figure 4. Endosomal Distribution of V2R. Live-cell confocal imaging of serum-starved HEK293 cells co-expressing SNAP-V2R and Tdr-rGFP-tagged endosomal markers: (A) Rab4, (B) Rab5, (C) Rab7, or (D) Rab11. Cells were stimulated with 100 nM AVP for 20 min at 37°C. Arrows indicate representative sites of colocalization between V2R and Rab-marked endosomes. Scale bars, 10 μm.

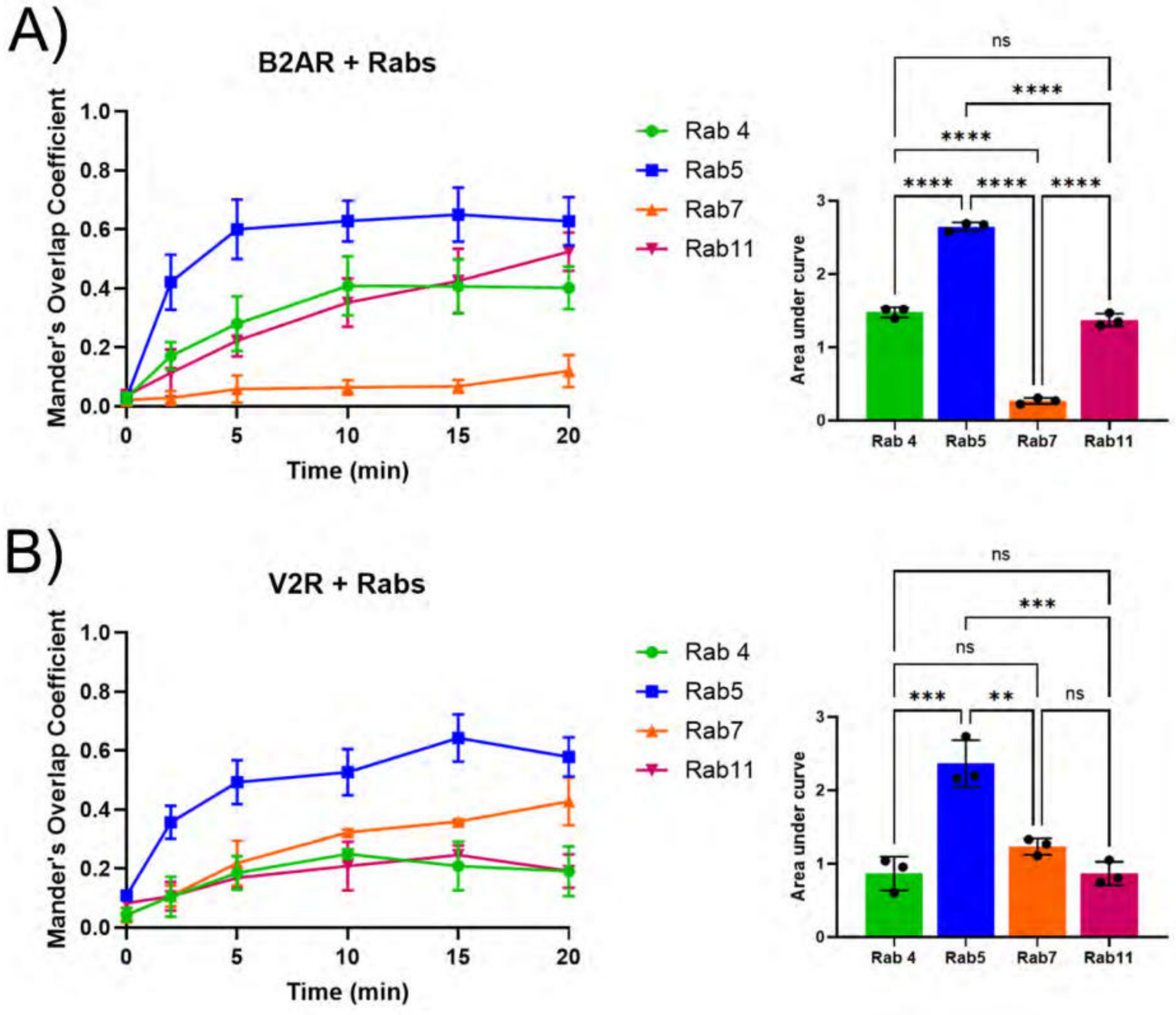

Supplementary Figure 5. Quantification of GPCR Colocalization with Endosomal Rab Markers. Colocalization between β2AR or V2R and endosomal markers (Rab4, Rab5, Rab7, Rab11) was quantified using Mander's overlap coefficient, based on confocal images shown in Supplementary Figures 3 and 4. Error bars indicate mean ± S.D. from n = 3 independent experiments. A total of 30–40 cells were analyzed per condition, with the same cells tracked over time (0–20 min stimulation). XY plots depict the time course of colocalization following stimulation with (A) β2AR (10 μM Iso) or (B) V2R (100 nM AVP). Corresponding histograms show the area under each curve. Statistical significance was assessed using one-way ANOVA followed by Tukey's multiple comparison test (*p < 0.05; **p < 0.01; ***p < 0.001; ****p < 0.0001; ns, not significant).

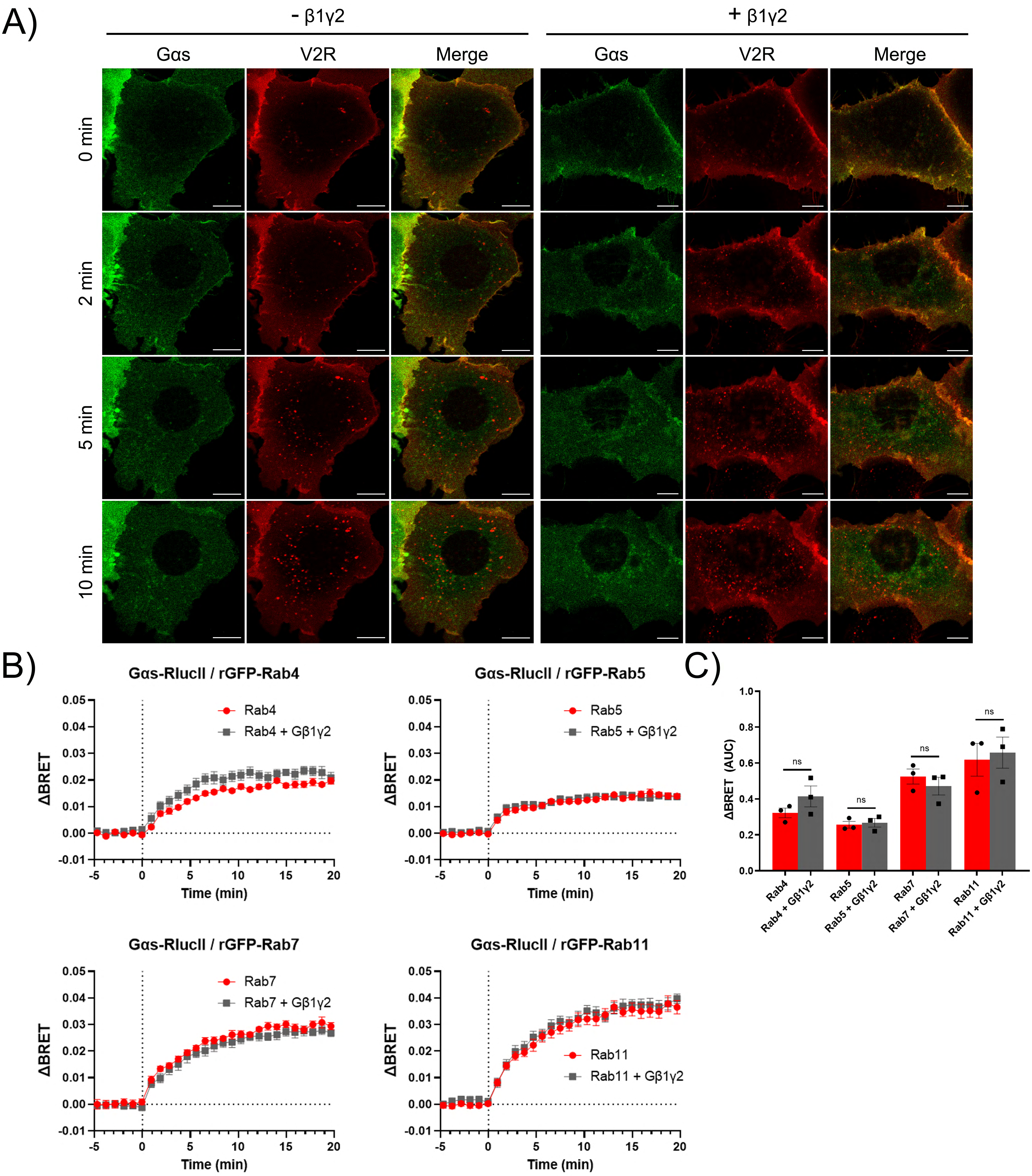

Supplementary Figure 6. Gβγ Overexpression does not Significantly Affect Gαs Trafficking. A) Serum-starved HEK293 cells co-expressing SNAP-V2R and Gαs-EGFP, with or without Gβ1 and Gγ2 overexpression, were stimulated with 10 μM Iso for 10 min at 37°C and imaged by live-cell confocal microscopy. Scale bars, 10 μm. (B) Time course of Gαs translocation to Rab4-, Rab5-, Rab7-, and Rab11-labeled endosomes following V2R activation with 1 μM AVP, monitored using the ebBRET biosensor assay described in Fig. 5A, in the presence or absence of Gβ1 and Gγ2 overexpression. The vertical dotted line marks agonist addition. Data are presented as normalized BRET values (ΔBRET), mean ± SEM of triplicates; N = 3. C) Quantification of agonist-induced BRET responses shown in (B) by calculating area under the curve (AUC) for each experiment. Statistical significance was assessed using Welch's t test; ns, not significant.

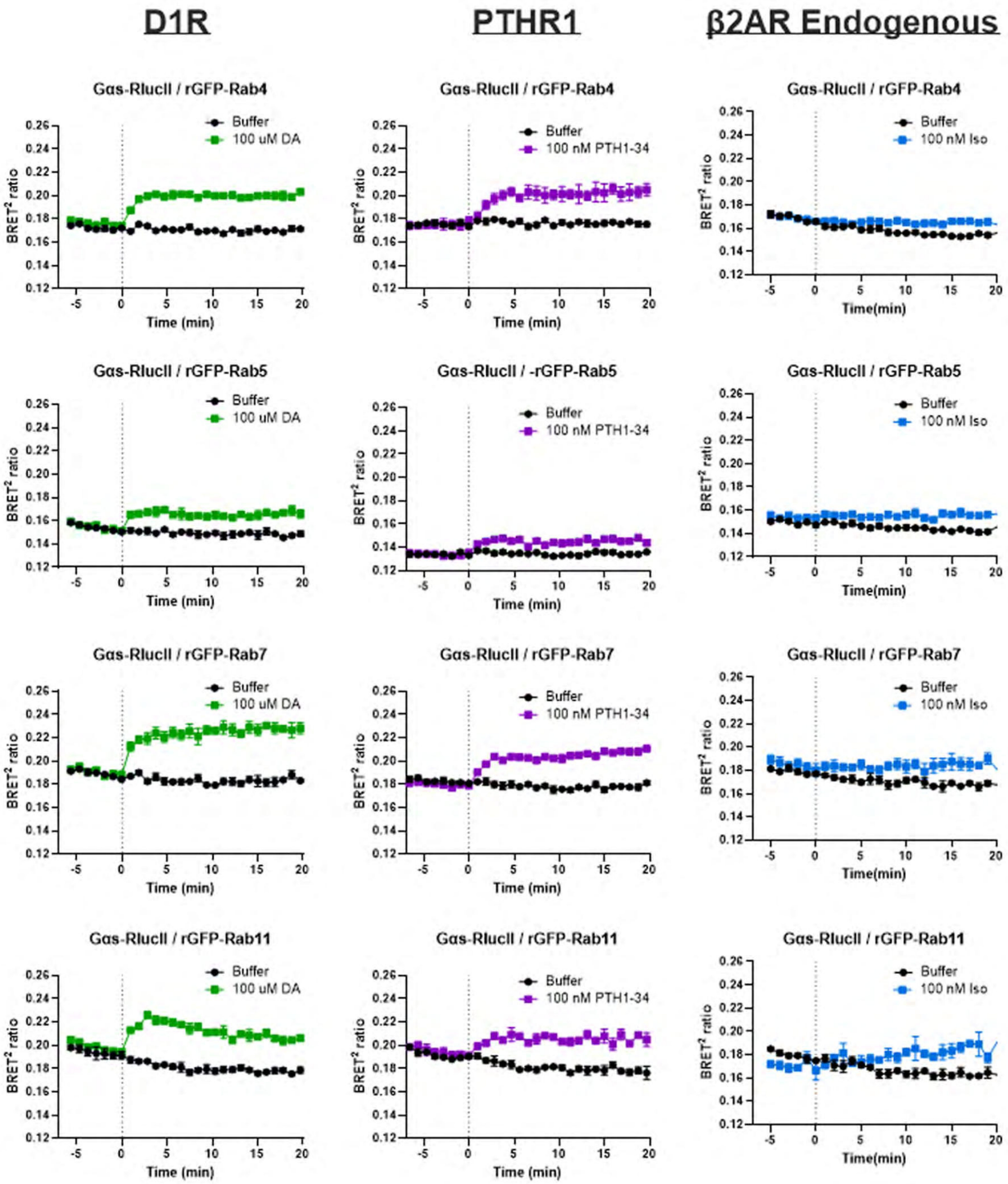

Supplementary Figure 7. Gαs Recruitment to Different Endosomal Compartments Following D1R, PTHR1 or Endogenous β2AR (complementary to Figure 5C). Time courses of Gαs translocation to Rab4-, Rab5-, Rab7-, and Rab11-labeled endosomes were measured following stimulation with 100 µM dopamine (D1R agonist), 100 nM PTH1-34 (PTHR1 agonist), or 1 µM Iso (endogenous β2AR) using the ebBRET biosensor assay described in Figure 5A. The vertical dotted line indicates the timing of agonist addition. Data are presented as mean ± SEM of triplicates from a representative experiment; experiments were performed three times with similar results.

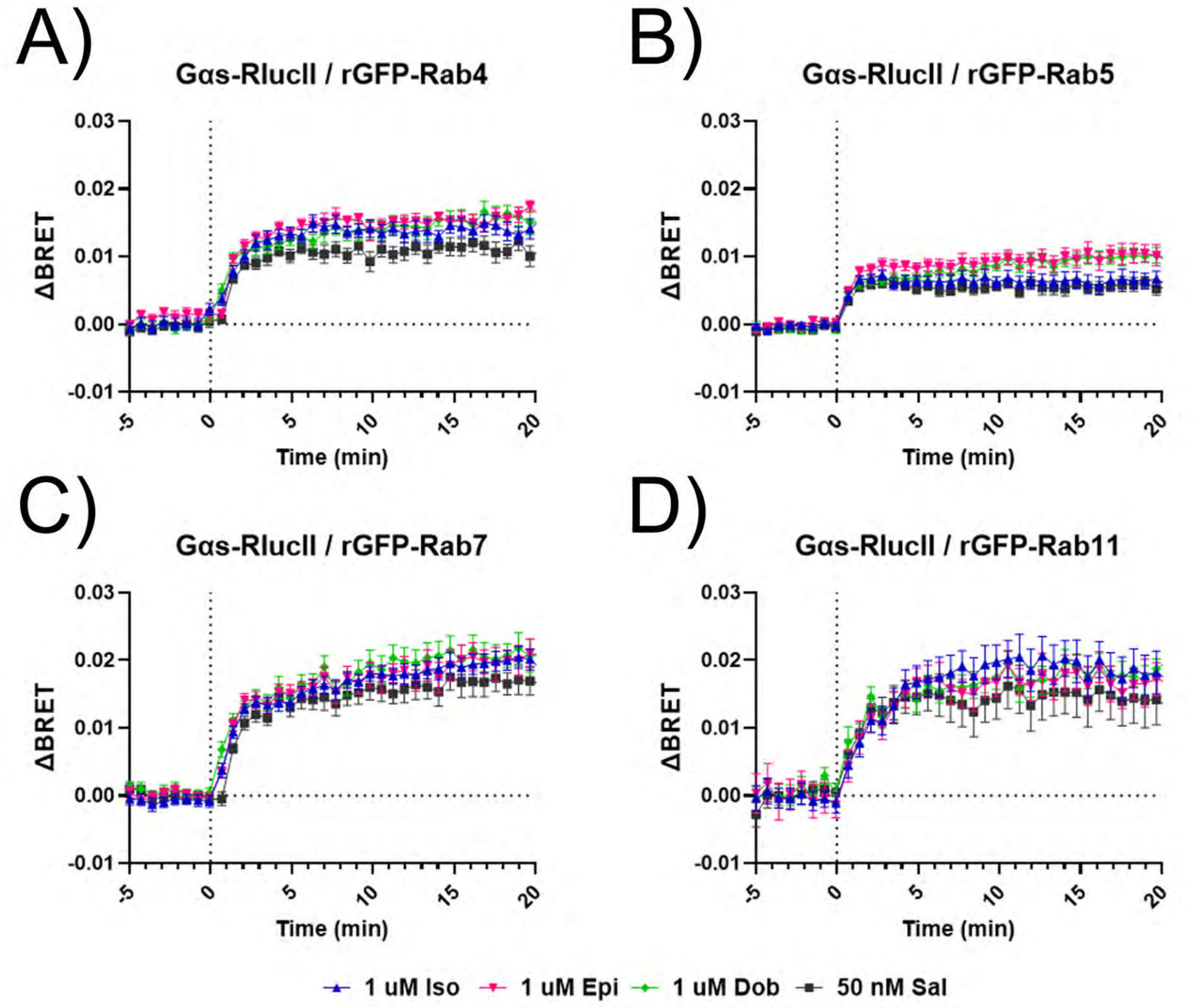

Supplementary Figure 8. Gαs Relocalization to Different Endosomal compartments Following β2AR Stimulation with Various Agonists. Time courses of Gαs translocation to Rab4-, Rab5-, Rab7-, and Rab11-labeled endosomes were measured following β2AR activation with different agonists, using the ebBRET biosensor described in Fig 5G. The vertical dotted line marks agonist addition. Data are shown as normalized BRET values (ΔBRET), presented as mean ± SEM of triplicate measurements; N = 4.

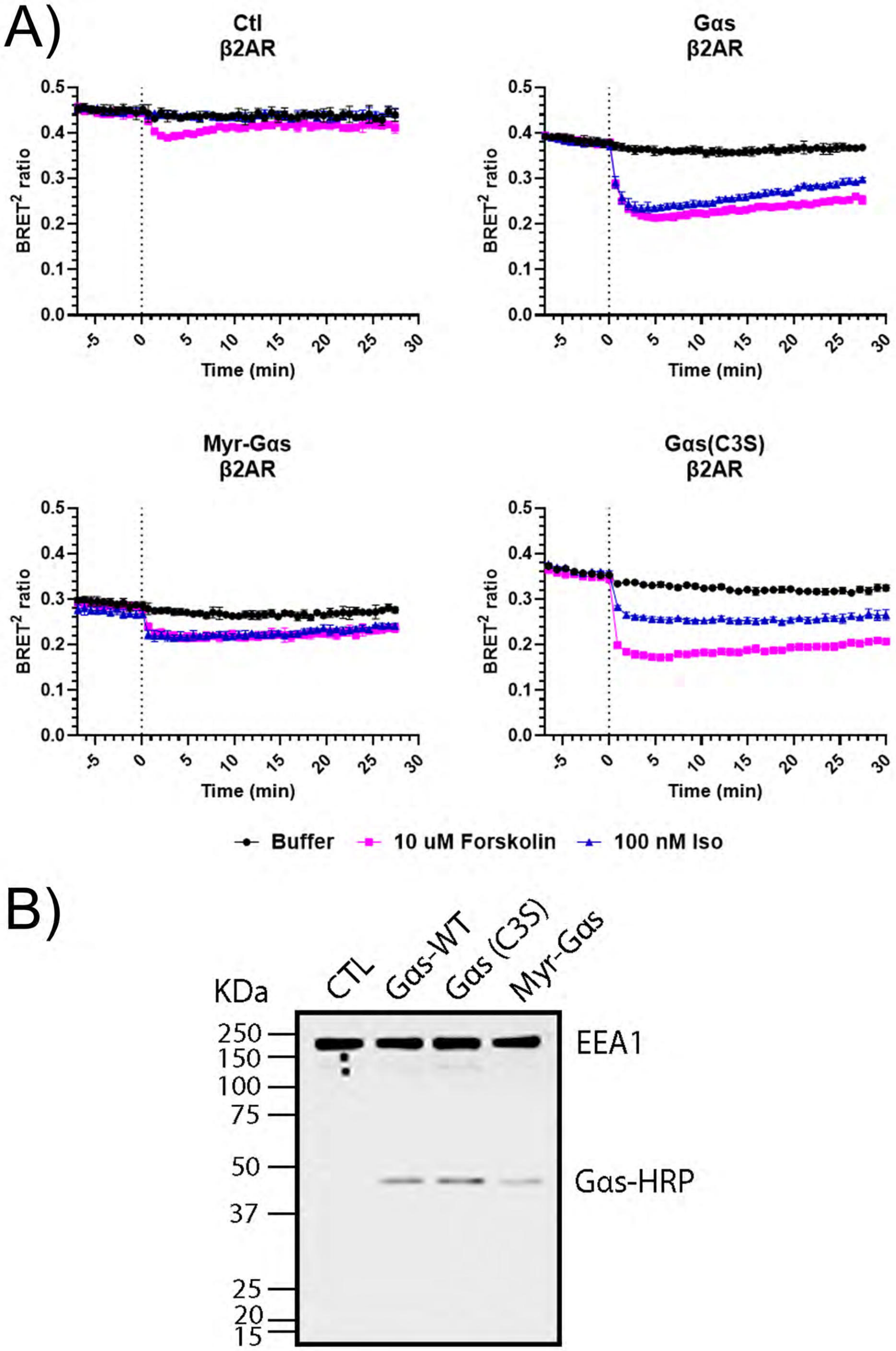

Supplementary Figure 9. Adenylate Cyclase Activation by Myr-Gαs and Gαs(C3S) Following β2AR Stimulation. (A) cAMP production in HEK-GαsKO cells was measured following β2AR stimulation with 100 nM isoproterenol (Iso), using the EPAC biosensor as described in Methods. Cells were transfected with β2AR alone or together with wild-type Gαs, Myr-Gαs, or Gαs(C3S). Forskolin served as a positive control for cAMP production. The vertical dotted line indicates the time of agonist addition. Data are presented as mean ± SEM of triplicates from a representative experiment; experiments were repeated three times with similar results. (B) Western blot showing expression levels of Gαs-WT, Gαs(C3S), and Myr-Gαs in samples corresponding to the ebBRET experiment shown in (A). EEA1 was used as a loading control.

Table S1. Primer Used.

Primer	Sequence	
Gαs-EGFP (Gαs)	Fwd:	5'- GATGGTGAGAAGGCAACC -3'
	Rev:	5'- GCCCTCTCCATTAAACCC -3'
Gαs-EGFP (EGFP)	Fwd:	5'- ATGGGTTTAATGGAGAGGGCTCCGGCGGAGGAGGAAGCATGGTGAGCAAGGGCGAG -3'
	Rev:	5'- TTGGTTGCCTTCTCACCATCGCTGCCACCACCTCCGGACTTGTACAGCTCGTCCATGC -3'
Gαs-mini HA	Fwd:	5'- GGCGGCGAAGAGGACGTCCCCGATTACGCTAGCAACAGCGATGGT -3'
	Rev:	5'- ACCATCGCTGTTGCTAGCGTAATCGGGGACGTCCTCTTCGCCGCC -3'
